# Airway immunometabolites fuel *Pseudomonas aeruginosa* infection

**DOI:** 10.1186/s12931-020-01591-x

**Published:** 2020-12-10

**Authors:** Sebastián A. Riquelme, Alice Prince

**Affiliations:** Department of Pediatrics, Columbia University, New York, NY 10032 USA

**Keywords:** *Pseudomonas aeruginosa*, Pneumonia, Succinate, Itaconate, Immunometabolism, Biofilm, Adaptation, Cystic fibrosis, ROS, Metabolic stress

## Abstract

Pulmonary infections are associated with a brisk inflammatory reaction to bacterial surface components. Lipopolysaccharides (LPS) trigger macrophage activation and release of mitochondrial metabolites that control the intensity of the immune response. Whereas succinate induces oxidative stress (ROS), HIF1α stabilization, glycolysis and IL-1β release, itaconate suppresses inflammation by inhibiting succinate oxidation, glycolytic flux and promoting anti-oxidant Nrf2-HO-1 functions. *P. aeruginosa* is a major pathogen associated with acute and chronic lung infection. Although both secreted toxins, LPS and proteases are key factors to establish acute *P. aeruginosa* pneumonia, lack of these components in chronic *P. aeruginosa* isolates suggest these organisms exploit other mechanisms to adapt and persist in the lung. Upon inhalation, *P. aeruginosa* strains trigger airway macrophage reprograming and bacterial variants obtained from acutely and chronically infected subjects exhibit metabolic adaptation consistent with succinate and itaconate assimilation; namely, high expression of extracellular polysaccharides (EPS), reduced *lptD*-LPS function, increased glyoxylate shunt (GS) activity and substantial biofilm production. In this review we discuss recent findings illustrating how *P. aeruginosa* induces and adapts to macrophage metabolites in the human lung, and that catabolism of succinate and itaconate contribute to their formidable abilities to tolerate oxidative stress, phagocytosis and immune clearance.

## Background

Opportunistic bacterial pathogens, such as *Pseudomonas aeruginosa*, *Klebsiella pneumoniae* and *Staphylococcus aureus* are frequently associated with persistent pulmonary infection [1, 2]. These pathogens are a major cause of morbidity and mortality, especially in individuals with damaged airways, as occurs in ventilator associated pneumonia (VAP) [3–6], in subjects with antecedent viral infection [7–10], or in patients exhibiting airway inflammation, as in chronic obstructive pulmonary disease (COPD) [11, 12] and in cystic fibrosis (CF) [13–16]. Antibiotic resistance is a common feature of these organisms, and may contribute to intractable infection, but even susceptible strains are able to cause chronic inflammation and eventual mortality, suggesting mechanisms other than drug resistance are involved in pulmonary pathogenesis. It is also curious that ex vivo, many of these bacteria are readily phagocytosed and killed by immune cells, suggesting that conditions within the airway itself, such as the complex metabolic milieu provided by inflammatory cells, may contribute to bacterial survival [1, 2]. The ability of these major opportunists to form biofilms, which protect bacteria from antibodies, complement, phagocytosis, antibiotic penetrance and especially from oxidants is a common factor in their pathogenicity and clearly contributes to their shared ability to cause persistent pulmonary infection [11, 17, 18]. Exactly what signals from the host activate the formation of biofilm are not well defined.

Biofilm formation results when a community of bacteria are able to form a nidus of infection on a surface, from an initial inoculum of planktonic organisms [19]. Biofilm-forming organisms attach to mucosal surfaces or form aggregates that are almost impossible for myeloid cells to engulf. This lifestyle is a defense response to oxidant stress [20] and can be triggered in vitro by reactive oxidant species (ROS) such as hydrogen peroxide (H_2_O_2_)[21, 22]. Biofilms generate a multilayered shield of extracellular polysaccharides (EPS), which are produced in response to both metabolic stress and structural damage to the bacteria. EPS functions as an oxidant sink [20, 23, 24], and the bacterial pathways that generate EPS components function to dissipate oxidant stress either by producing ROS-scavenging byproducts or by simply decreasing the metabolic rate to decrease endogenous ROS synthesis [25, 26]. The pathogenesis of *P. aeruginosa* infection in patients with CF provides an especially well-studied example of the development of pulmonary infection and biofilm formation in vivo in response to the pro-oxidant environment [15, 16]. While there have been numerous theories to explain the specificity of *P. aeruginosa* for the CF airways, there are ample data demonstrating that the infecting organisms form biofilms in vivo and that these bacterial communities are a major factor in the limitations of antimicrobial therapy in this disease [15, 27–29]. A prominent characteristic of the CF airway is the substantial accumulation of immune cells, phagocytes and T cells, which generate oxidant and inflammatory mediators that damage the lung [14]. These cells produce metabolites that determine the airway oxidant profile in response to *P. aeruginosa*. Such conditions select for bacteria with the metabolic plasticity that enables bacterial exploitation of these immune byproducts for the production of biofilms [2, 30].

CF is a genetic disease caused by mutations in the CF transmembrane conductance regulator (CFTR) that results in an altered accumulation of airway metabolites and ions, especially succinate, itaconate, chloride and bicarbonate [30, 31]. As these patients are typically followed from infancy, often before the establishment of pulmonary infection, through adulthood, they provide the unique opportunity to follow the adaptation of bacteria to the human airway over prolonged periods of time. This provides the opportunity to see how bacteria alter their own gene expression once they take up residence in the lung. The adaptation of *P. aeruginosa* to the CF lung provides insights into the host factors that initiate and promote chronic infection, as well as how tightly controlled the metabolic immune response must be to avoid excessive oxidant stress and *P. aeruginosa* biofilm development. In this review, we will explore how the accumulation of the immunometabolites succinate and itaconate provides a milieu that initially promotes the proliferation of *P. aeruginosa*, then generates selection for variants that produce abundant EPS instead of LPS, generates intractable biofilms and enables chronic infection.

## Main text

### Macrophage activation and inflammation in the infected airway

The human airway is replete with carbon sources for bacterial proliferation, essential microelements such as iron (Fe^++^) and nutrients like amino acids and lipids [32]. Ubiquitous opportunists, such as *P. aeruginosa* commonly found in the environment, colonizing sinks and showers as well as streams, are often inadvertently inhaled. The vast majority of these organisms are rapidly cleared, but those that persist must forcibly adapt to the microenvironment provided by the airway.

**Fig. 1:**
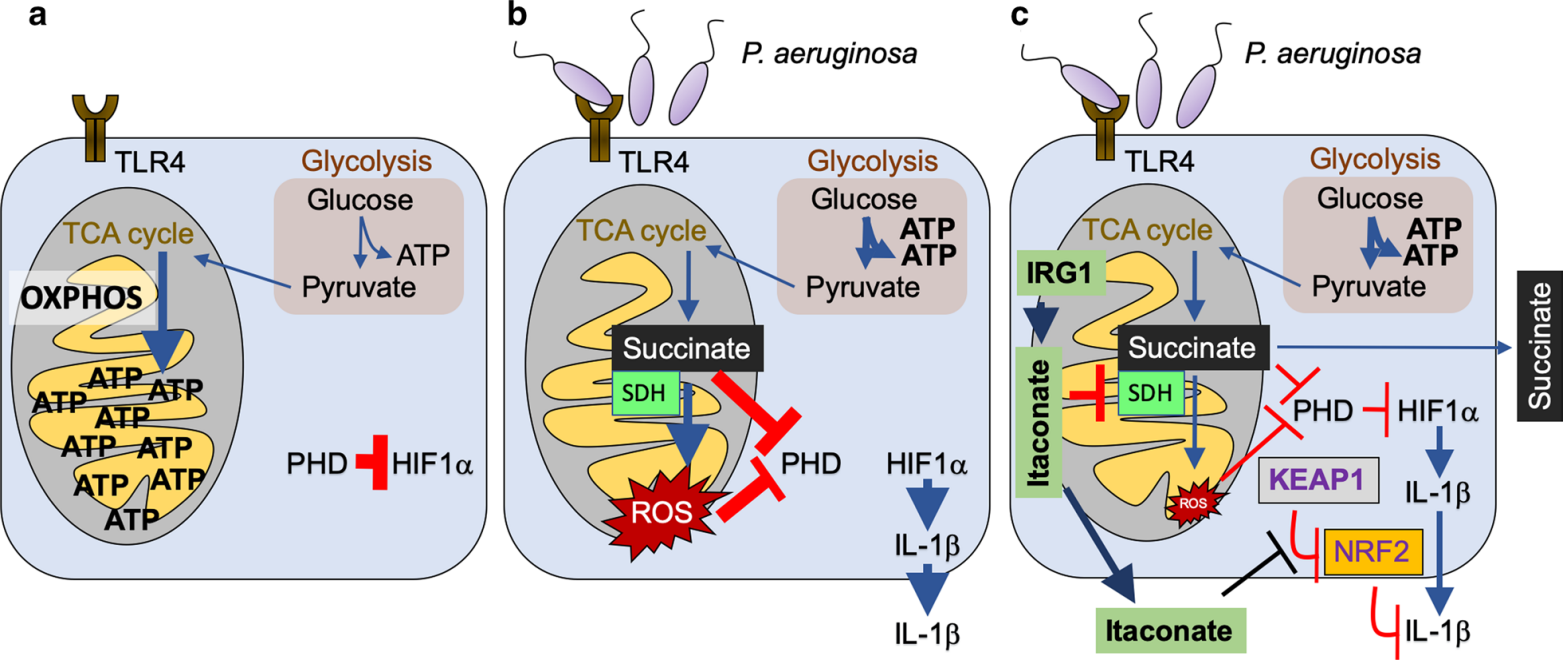
Immunometabolites succinate and itaconate are released during airway macrophage infection. **a** During homeostasis, airway macrophages employ the TCA cycle and OXPHOS to generate energy (ATP) in the mitochondria. In these conditions, the pro-inflammatory transcription factor HIF1α is inhibited in the cytoplasm by prolylhydroxylases (PHD). **b** Upon bacterial LPS detection by toll-like receptor 4 (TLR4), cells exhibit metabolic reprograming. Mitochondria become a major source for reactive oxygen species (ROS), which is mostly derived from succinate accumulation and its oxidation by succinate dehydrogenase (SDH). Succinate and ROS inhibits PHD, which release HIF1α to promote transcription of pro-inflammatory cytokines like IL-1β. Glycolysis becomes the major ATP source. **c** To avoid excessive tissue oxidation, macrophages also upregulate Immunoresponsive Gene 1 (IRG1), which synthetizes the SDH and KEAP1 inhibitor itaconate. SDH blockade induces succinate accumulation, which is excreted from the cell together with itaconate. Reduced SDH function by IRG1 regulates airway HIF1α and IL-1β activity. Itaconate is bactericidal

Much of what is known about the composition of airway fluid has been obtained from studies of CF patients, focusing primarily upon the concentrations of Na^+^, Cl^−^, HCO3^−^ and water in the setting of infection [32–34]. In studies using LPS as a surrogate for bacterial infection, macrophages very rapidly switch their resting metabolic activity from oxidative phosphorylation (OXPHOS) to glycolysis, with the resulting accumulation, oxidation and release of succinate and its byproduct ROS [35–38]. Both metabolites activate the hypoxia induce factor 1α (HIF-1α) and IL-1β production initiating a proinflammatory response [35–37] (Fig. 1a, b), which is associated with the recruitment and activation of phagocytes. Thus, IL-1β is an important proinflammatory cytokine expected to facilitate the eradication of *P. aeruginosa*. However, in several models of pneumonia, IL-1β, in fact, contributes to *P. aeruginosa* infection [39–41]. Mice lacking a receptor for IL-1β (*il1r*^*−/−*^) or those treated with its antagonist Anakinra actually clear *P. aeruginosa* significantly better than those with a robust IL-1β response. The inflamed CF airway with many activated macrophages and monocytes is enriched for succinate, in comparison to normal control broncheoalveolar lavage (BAL) fluid, and elevated amounts of succinate have also been observed in animal models mimicking the CF disease [30, 42, 43] (Fig. 1a, b). Thus, an important substrate for bacterial proliferation, succinate, is immediately available for bacterial consumption upon their entry into the airway, and is provided by activated myeloid cells.

### Succinate is a preferred carbon source for environmental *P. aeruginosa*

**Fig. 2:**
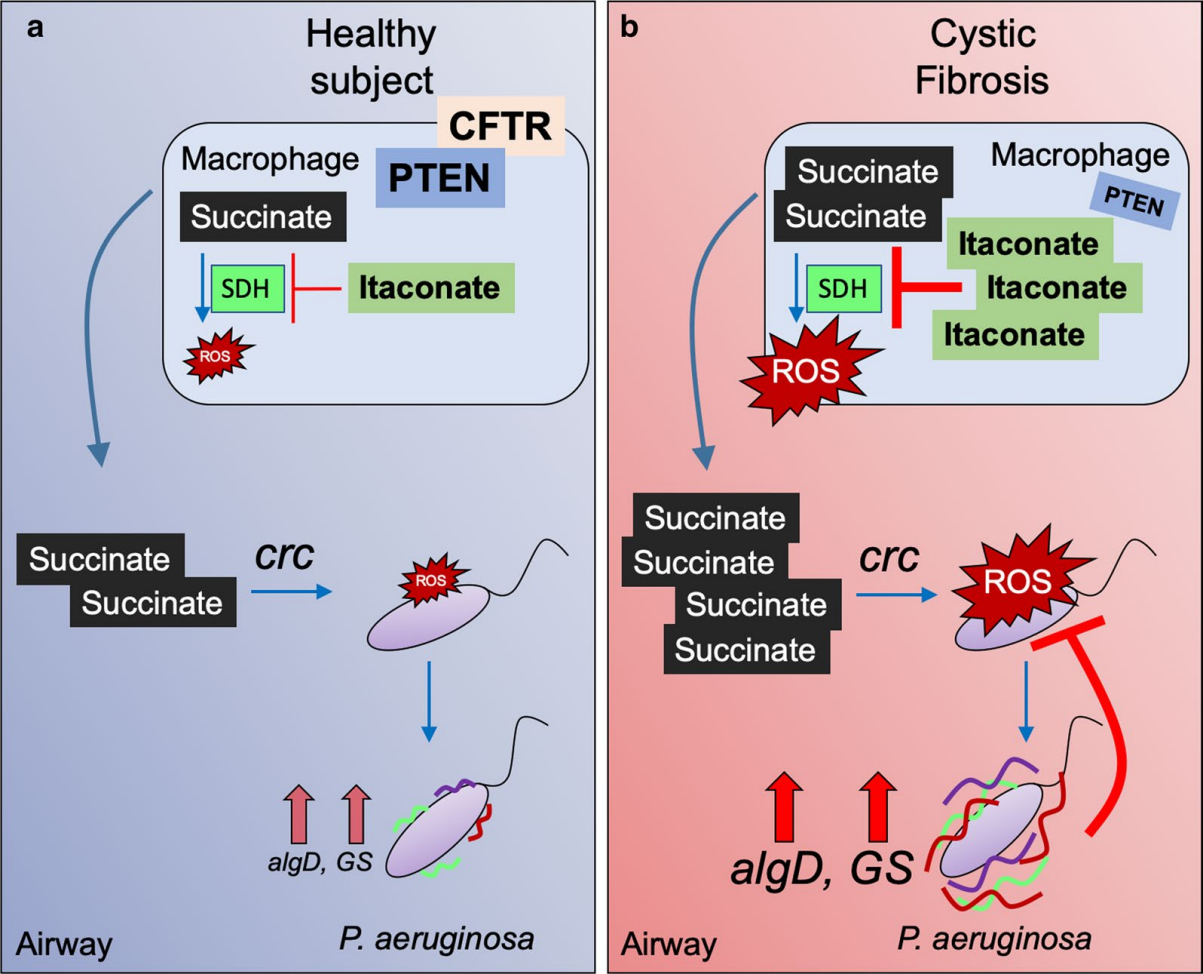
The CFTR-PTEN complex controls airway succinate accumulation and *P. aeruginosa* metabolic reprograming. **a** In healthy subjects, the CFTR-PTEN complex controls the amount of succinate released into the airway during *P. aeruginosa* infection. The catabolite control repressor *crc* locus induces succinate assimilation and its oxidation in *P. aeruginosa*. These succinate levels are associated neither with high oxidative stress nor selection of *P. aeruginosa* strains overexpressing *algD* or anti-oxidant glyoxylate shunt (GS) components. **b** In cystic fibrosis (CF) subjects, lack of the CFTR-PTEN complex compromises the oxidant response to infection promoting increased succinate release into the airway. These high succinate levels induce more *crc* activity, which is associated with elevated oxidative stress in *P. aeruginosa*. Succinate-stressed *P. aeruginosa* overexpresses the anti-oxidant *al*g*D* and GS components, which bypasses the pro-oxidant TCA cycle to reduce internal ROS production. These strains are more protected from oxidative stress and form biofilm in response to succinate. Color lines on *P. aeruginosa* are extracellular polysaccharides, such as *algD*-mediated alginate

Many different types of inhaled bacteria are likely to be entrapped by the dehydrated airway secretions that are prominent in CF; but in fact, only a few species cause persistent infection. These opportunists, most prominently *P. aeruginosa* must rapidly adapt to the available metabolites, including macrophage succinate. In contrast to many other bacteria that prefer glucose or amino acids, *P*. *aeruginosa* preferentially consumes succinate as directed by its *crc* locus, before utilizing other carbon sources [44–48] (Fig. 2a). However, forced succinate consumption generates substantial oxidant stress for the organisms, which are already in an oxidant rich environment with activated phagocytes releasing ROS [14, 20, 24, 48]. As a response, *P. aeruginosa* variants are selected that utilize the anti-oxidant glyoxylate shunt (GS) to dissipate ROS and to generate biofilm, which itself has anti-oxidant properties [25, 26, 30, 49].

Laboratory strains of *P. aeruginosa* grown in high levels of succinate demonstrate metabolic changes that enable them to proliferate amidst high levels of oxidants [30] (Fig. 2b). These bacteria induce even more succinate in the airway as well as myeloid cell death, consistent with increased secretion of IL-1β and pyroptosis [30]. Succinate-exposed strains increased their glucose metabolism and utilization of threalose and acetate, which feed the GS, production of EPS and enable tolerance to oxidant stress. *P. aeruginosa* grown in high succinate were phenotypically different, with increased colony size, consistent with the abundant production of EPS. These strains caused significantly greater levels of infection in mouse models of pneumonia, decreased myeloid cell viability and promoted more IL-1β release. Importantly, the same constellation of metabolic and anti-oxidant changes was identified in a collection of clinical *P. aeruginosa* strains cultured from an adult with CF [30, 50, 51]; namely, altered carbon substrate utilization, increased use of the GS and expression of genes associated with EPS and biofilm formation. Both the clinical strains from CF and the *P. aeruginosa* grown in high succinate in vitro were more proficient in colonizing the airways of mice, causing persistent infection that lasted for days. Thus, the ability to metabolize succinate and modify their own metabolic activity in response to the ROS generated by this immunometabolite enables *P. aeruginosa* to proliferate and adapt to the pro-oxidant airway environment.

### Airway succinate is regulated by phosphatase and tensin homologue deleted on chromosome 10 (PTEN)

PTEN, by controlling the P3K/Akt/mammalian target of rapamycin (mTOR) pathway regulates cellular proliferation, glycolytic metabolism [52] and mitochondrial activity [53–55]. PTEN participates in the regulation of mitochondrial respiration, especially controlling the function of cytochrome C[54] and the assimilation of the oxidative TCA cycle substrate isocitrate [30]. PTEN dysfunction induces increased expression of isocitrate dehydrogenase (IDH), which overstimulates mitochondrial complex I by providing with more nicotinamide adenine dinucleotide (NADH)[30]. Increased complex I activation induces more oxygen reduction and ROS, which, during LPS stimulation, is reinforced by electrons reversely transferred from complex II, succinate dehydrogenase (SDH)[37]. This excessive ROS production activates the anti-oxidant cell response, which, in part, is mediated by itaconate. This metabolite is the product of *Irg1* (in humans *Acod1*), and its activity is linked to, for example, inhibition of SDH [56–59]. Thus, PTEN deregulation induces oxidative stress in mitochondria and, as a compensatory mechanism, itaconate synthesis, which inhibits SDH and enable succinate accumulation and its release [30, 60]. For proper PTEN metabolic activity, this phosphatase associates with the C-terminal cytoplasmic tail of CFTR, which is known to increase its stability and activation by dephosphorylation [61]. In cells (and patients) with decreased membrane-associated CFTR, or in subjects harboring mutations in the CFTR C-terminal tail, there is also decreased PTEN numbers and increased succinate accumulation [42, 61] (Fig. 2). This deregulation is associated with more airway inflammation [61, 62]. *P. aeruginosa* infection of myeloid and epithelial cells induces both PTEN reduction and succinate release, and peripheral blood mononuclear cells (PBMCs) from individuals exhibiting CFTR-PTEN deficiency secrete much more succinate levels than controls after infection with these organisms [30, 61]. The airway of CFTR-PTEN deficient subjects showed tenfold succinate accumulation respect with healthy individuals, demonstrating that the metabolic activation status of epithelial and myeloid cells can impact the pro-oxidant composition of the airway metabolome. As the CFTR-PTEN association is not dependent upon the channel function of CFTR, therapeutic strategies that increase the membrane localization of CFTR should also increase PTEN and normalize succinate, inflammation and also reduce *P. aeruginosa* infection.

### Itaconate is an immunometabolite that protects the airway from oxidative damage

**Fig. 3:**
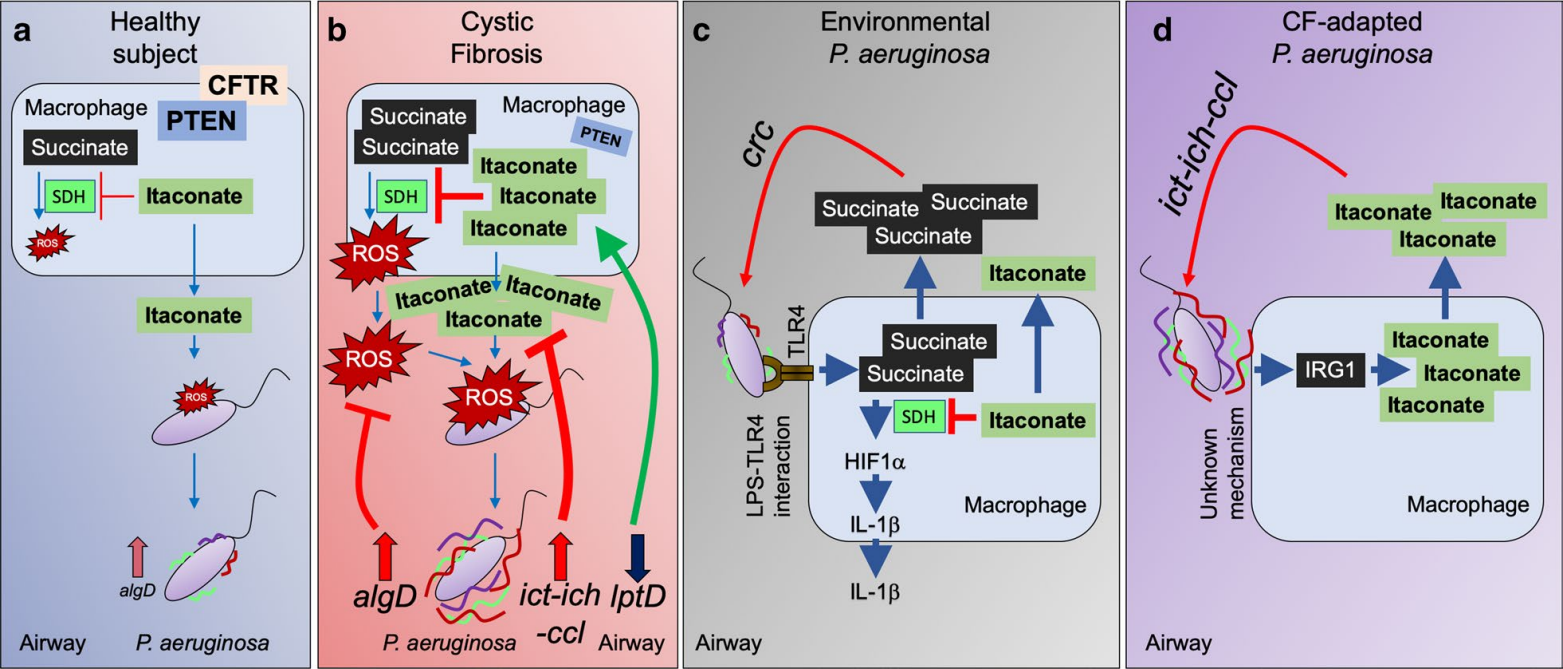
Itaconate fuels *P. aeruginosa* adaptive changes and chronic infection. **a** In healthy subjects, itaconate is produced during *P. aeruginosa* infection to control SDH activity, oxidative stress and inflammation. This itaconate levels are tolerated by *P. aeruginosa* during acute infection. **b** In CF individuals lacking CFTR-PTEN complex activity, elevated succinate oxidation induces synthesis and release of the anti-oxidant molecule itaconate as a compensatory mechanism. Airway itaconate induces *P. aeruginosa* outer membrane stress, which induces *ict-ich-ccl* locus overexpression to degrade itaconate. Itaconate also induces downregulation of *lptD*, which suppresses surface exposure of LPS. Lack of surface-exposed endotoxin causes bacterial membrane deregulation and permeability, which is compensated by activation of the *algT-algD* membrane stress response to produce more protective alginate. Through an unknown mechanism, alginate induces more itaconate release by host macrophages, which fuels biofilm production, adaptation and long-term infection. **c** Environmental *P. aeruginosa* strains expressing LPS induce the TLR4-succinate-HIF1α-IL-1β axis, inducing release of succinate and regulatory itaconate. Succinate released fuels *P. aeruginosa* infection through the *crc* locus during acute infection. **d** Host-adapted *P. aeruginosa* isolates, which lack surface LPS and overproduce alginate, induce IRG1 expression and high itaconate production in macrophages. IRG1 induction is mediated by alginate. Itaconate released fuels host-adapted *P. aeruginosa* through the *ict-ich-ccl* locus activity. Color lines on *P. aeruginosa* are extracellular polysaccharides, such as *algD*-mediated alginate

Itaconate, an electrophilic carboxylate, is also a prominent metabolite found in the infected airway [30, 59] (Figs. 1c, 3). As described above, itaconate is released by LPS activated macrophages in response to succinate oxidation by SDH, and functions to counter the proinflammatory response caused by ROS and IL-1β [63]. Itaconate and its derivatives 4-octyl itaconate and dimethyl-itaconate suppress inflammation by targeting Kelch-like ECH-associated protein 1 (KEAP1), which sequesters the anti-oxidant Nuclear Factor–Erythroid-2–Related Factor 2 (NRF2) transcription factor in the cytoplasm [64, 65]. Itaconate alkylates KEAP1, releasing NRF2 from proteosomal degradation and promoting its migration to the nucleus where it induces expression of multiple anti-oxidant genes such as HMOX1 (Heme-oxygenase 1), as well as suppress production of IFNβ [64]. Itaconate also suppresses secretion of inflammasome associated pro-inflammatory cytokines by dicarboxypropylation of pyrin domain-containing protein 3 (NLRP3), which reduces its interaction with NEK7 [66]. Reduced NLRP3-NEK7 complex formation abolishes the LPS-induced assembly of the inflammasome, preventing the production of, for example, IL-1β, and in PBMCs from subjects exhibiting the IL-1β based disease cryopyrin-associated periodic syndrome (CAPS) itaconate and 4-octyl itaconate reduce IL-1β production.

During Gram-negative infection of mucosal surfaces, itaconate participates in the metabolic control of inflammation by suppressing release of pro-inflammatory cytokines by interrupting the oxidation of succinate by SDH [63, 67]. Reduced transformation of succinate into fumarate diminishes the amounts of ROS required to inhibits prolyl hydroxylases (PHD), and impeding HIF1α migration to the nucleus to promote transcription of pro-glycolytic and pro-inflammatory genes [35]. Reduced SDH function due to itaconate accumulation safeguards the integrity of the mucosal tissue, as it induces less local damage and accelerates the process of tissue repair. Itaconate also interacts with macrophage aldolase, where it induces cysteine modifications that reduce its catalytic function and, eventually, inactivating glycolytic flux and the release of effector cytokines that might harm the lung [68]. *Irg1* null mice are highly susceptible to the hyperinflammatory pathology associated with LPS [63]. In other pulmonary infections, the lack of itaconate enables massive recruitment of myeloid cells to the *Mycobacterium tubercul*osis infected lung, exacerbating inflammation, disrupting pulmonary function and reducing mouse survival [69]. *Irg1* deficiency is associated with greater amounts of IL-1β accumulation in airway of mice infected with laboratory strains of *P. aeruginosa *[59], confirming the anti-inflammatory and protective role this metabolite has in the setting of pulmonary infection. The abundance of both anti—inflammatory itaconate and pro-inflammatory succinate in the airway suggests that successful pathogens adapt to both macrophage metabolites in order to persist.

### *P. aeruginosa* induces and consumes airway itaconate

In contrast to other organisms more susceptible to the electrophilic stress imposed by itaconate, such as *Legionella pneumophila*, *S. aureus* and *Acinetobacter baumannii *[70], *P. aeruginosa* has adapted to this metabolite, and are forced to degrade itaconate to survive. *P. aeruginosa* expresses 3 genes (*ich*, *ict*, *icl*) devoted to itaconate metabolism, enabling them to assimilate itaconate in the airway as carbon source [59, 70, 71] (Fig. 3a, b). This is a property shared by *M. tuberculosis* and the *Aspergillus* species [71, 72], suggesting an evolutive and conserved mechanism of adaption to host immunometabolites. The consumption of itaconate by the *ict-ich-ccl* locus generates acetyl-coA and pyruvate, which are feeders of the GS and TCA cycle, respectively and provide the components for biofilm production.

Clinical isolates of *P. aeruginosa* adapt to itaconate in vivo, with increased expression of the *ict-ich-ccl* locus, and induction of biofilm production. The clinical strains shift their preferred airway carbon source from succinate to itaconate. Itaconate stress causes increased bacterial outer membrane permeability, reducing the capacity of *P. aeruginosa* strains to transport LPS to their surface. These outer barrier function changes activate the *algT*-*algD*-MucA membrane stress repair system [73, 74], facilitating the replacement of LPS with more protective alginate polymers. Itaconate exposed laboratory strains of *P. aeruginosa* have diminished production of O-side chains of LPS [59], confirming that these organisms divert carbohydrates from endotoxin synthesis to the generation of EPS, in response to membrane stress. These same findings were apparent in clinical isolates of *P. aeruginosa* from CF patients, which had been chronically exposed to airway itaconate. These CF strains had developed mutations in the pathways involved in LPS surface display, specifically in the *lptD* locus [59]. LptD is the LPS transporter embedded in the outer membrane that flips and anchors the endotoxin towards the extracellular side [75, 76]. *P. aeruginosa* CF isolates from chronic infection fail to expose LPS nor do they induce macrophage release of succinate, as do environmental strains of *P. aeruginosa*. Of note, the EPS (alginate) produced by these isolates stimulates itaconate release from host immune cells [59]. The mechanisms by which *P. aeruginosa* EPS induces macrophage itaconate remains unclear. The changes in the display of EPS versus LPS on the surface of *P. aeruginosa* in chronic airway infection suggests that these organisms are forced to catabolize itaconate to prevent outer membrane disruption and biofilm clearance. The capacity of the *ict-ich-ccl* locus to provide *P. aeruginosa* with carbon structures such as acetyl-coA and pyruvate demonstrate its valuable contribution to the establishment of long-term lung colonization.

### Immune signaling activated by itaconate-adapted *P. aeruginosa*

It is well appreciated that the induction of EPS moieties and biofilm production, especially the alginate overproducers characteristic of chronic infection in CF, correlate with the onset of intractable pulmonary infection [77]. Thus, the selection of these variants that induce itaconate production and then consume the metabolite, is of considerable clinical significance. The bacterial adaptive response, the production of EPS contributes to accumulation of phagocytes in the airways, but without resulting in bacterial clearance [78]. The prevailing dogma is that organisms enveloped in EPS, such as those with increased *algD* or *pslA* resist phagocytosis and are “less” immunogenic [24, 79, 80]. However, when itaconate-adapted clinical strains were examined for immunogenicity either in vivo or in vitro, they readily induced recruitment of immune cells into the airway [30, 59]. All of the strains stimulated variable amounts of IL-6 and TNFα, and recruited greater numbers of neutrophils and monocytes than control bacteria [30, 59]. The activation and release of substantial amounts of IL-1β was limited to the laboratory strain PAO1, perhaps due to its expression of LPS, flagella and toxins that directly activate the inflammasome and generate the release of succinate [39, 81] (Fig. 3c). The CF strains had lost expression of pathogen-associated molecular patterns (PAMPs) that are expected to induce IL-1β, specially LPS, flagella and the type 3 toxins [2, 16, 30, 59, 82], which was consistent with their failure to activate the release of succinate [35, 36] (Fig. 3d).

The immunogenicity of these *P. aeruginosa* CF isolates differed from that of the LPS-expressing laboratory strains, which have never been exposed to the itaconate-rich airway before [30, 59] (Fig. 3d). Although these CF strains failed to induce IL-1β, they stimulated myeloid cell reprograming generating itaconate. In sputum from CF patients infected with host-adapted *P. aeruginosa*, there is substantial accumulation of monocytes and macrophages that release itaconate. These findings indicate that the abundance of the immunometabolite itaconate has a major role in the selection of *P. aeruginosa* variants, promoting the display of EPS and not LPS on their surfaces and further contributing to the accumulation of itaconate and the establishment of intractable biofilms.

## Conclusions

The availability of specific carbon sources in the airway, specifically, the relative amounts of succinate and itaconate are not typically considered major factors in susceptibility to infection. Instead, the relative resistance of bacteria to antibiotics, susceptibility to phagocytosis, and expression of toxins, either leukocidins or destructive proteases are typically considered the major factors promoting pulmonary infection [83–88]. However, it is important to consider how specific pathogens adapt to the airway and adjust their own metabolism to the biofilm mode of growth that promotes persistent infection. Historically, bacteria have been speciated according to their metabolic preferences, a methodology now supplanted by genomic studies, but remarkably useful in classifying specific organisms and their clinical relevance. Hence, *P. aeruginosa* has been classified as a “non-lactose fermenter” putting it in a selective category of opportunistic Gram-negative pathogens. As reviewed here, bacterial substrate preference turns out to be critical, first in the initial infection of the airway and then in the selection of proficient biofilm formers.

Both planktonic and biofilm associated bacteria persist over the course of infection, but at some point, the host-adapted strains predominate and an itaconate-dominant immuno-metabolic milieu is generated. Thus, the nature of the immunometabolites that are released during the course of *P. aeruginosa* infection and the multiple PAMPs that induce their excretion are a major factor in the success of this pathogen as a cause of chronic pulmonary infection. Further studies are needed to elucidate how *P. aeruginosa* and many other respiratory pathogens induce and exploit particular immunometabolic responses to cause acute and then transition into long-term disease. The characterization of these pathways would provide with new targets to control airway infections, such as identification of which metabolites released by host macrophage fuel toxin production, biofilms, development of antibiotic resistance and promotion of adaptive changes.

The ability of *P.aeruginosa* to exploit the production of immunometabolites that are produced as a component of host defense clearly adds to their success as pulmonary pathogens. Moreover, the infecting organisms display a range of metabolic activities, as well illustrated by the phenotypic heterogenicity of the *P. aeruginosa* isolates in the CF airway, ranging from mucoid [15, 78] to small colony variants [29], each of which exhibit distinct metabolic profiles. The efficacy of antimicrobial therapy against *P.aeruginosa* infection might be complemented by targeting some of these metabolic pathways that are activated in vivo, such as itaconate degradation by the *ict-ich-ccl* locus [59]. Inhibition of this protective response might render *P. aeruginosa* susceptible to itaconate toxicity, reducing bacterial loads and their ability to produce biofilm. In parallel, administration of compounds that block *P. aeruginosa* assimilation of succinate might prevent their adaptation and synthesis of EPS. By forcing the organisms to maintain a planktonic lifestyle, phagocytic clearance would be improved. Better understanding of the bacterial machinery, activated in vivo, that function to transport these metabolites would also be useful. Competitive inhibitors to selectively block bacterial uptake of succinate and itaconate from the environment would limit the ability of this pathogen to proliferate in the airway.

An alternative therapeutic approach could target host metabolism. Incorporation of exogenous PTEN into the CF mitochondria, which would reduce the accumulation of immunometabolites in the infected airway, is another approach that merits further investigation [30]. It is challenging to deliver this phosphatase inside airway cells in the CF lung, although its recombinant isoform PTEN-long can be successfully internalized into tumor cells due to its poly-cationic N-terminal domain [89]. As the currently available CFTR potentiator and corrector therapies, by increasing membrane associated CFTR, will also increase PTEN function [61], further suppressing the accumulation of succinate and itaconate may be very effective in limiting *P. aeruginosa* adaptation and persistence. The complex association between the airway immune-metabolic responses to infection and *P. aeruginosa* suggest that this approach might be successful.

## Data Availability

This manuscript does not include data and material than can be shared.

## Abbreviations

CFTR: Cystic fibrosis conductance transmembrane regulator
PTEN: Phosphatase and tensin homologue deleted on chromosome 10
ROS: Reactive oxygen species
IRG1: Immunosuppressive gene 1
SDH: Succinate dehydrogenase
NRF2: Nuclear factor–erythroid-2–related factor 2
HO-1: Hemeoxygenase 1
KEAP1: Kelch-like ECH-associated protein 1
TLR4: Toll-like receptor 4
LPS: Lipopolysaccharide
EPS: Extracellular polysaccharide
HIF1α: Hypoxida induced factor 1α
COPD: Chronic obstructive pulmonary disease
CF: Cystic fibrosis
NLRP3: Pyrin domain-containing protein 3
NADH: Nicotinamide adenine dinucleotide
IDH: Isocitrate dehydrogenase
